# 4-Chloro-*N*-(3-chloro­phen­yl)benzamide

**DOI:** 10.1107/S1600536809034308

**Published:** 2009-09-12

**Authors:** Susanta K. Nayak, M. Kishore Reddy, T. N. Guru Row

**Affiliations:** a Solid State and Structural Chemistry Unit, Indian Institute of Science, Bangalore 560 012, Karnataka, India

## Abstract

The title compound, C_13_H_9_Cl_2_N, has an intra­molecular C—H⋯O  close contact, and presents the NH group *syn* to the *meta*-chloro group in the aniline ring and *trans* to the C=O group. The crystal packing is formed by infinite chains of N—H⋯O hydrogen bonds along the *c* axis. Cl⋯Cl [3.474 (1) Å] contacts link chains. The crystal used for data collection was a twin, the domains related by the twin law 0.948 (1)/0.052 (1).

## Related literature

For halogen inter­actions in the benzanilide series, see: Chopra & Guru Row (2005 ▶, 2008 ▶); Saeed *et al.* (2008 ▶); Gowda *et al.* (2008 ▶). For Cl⋯Cl inter­actions, see: Bui *et al.* (2009 ▶). For the program *ROTAX*, used to determine the twin law, see: Pearson & Gould (2003 ▶).
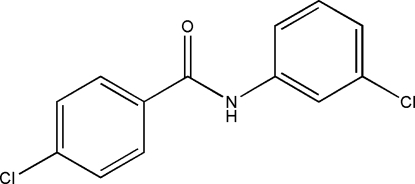


## Experimental

### 

#### Crystal data

C_13_H_9_Cl_2_NO
*M* *_r_* = 266.11
Monoclinic, 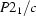

*a* = 12.8696 (15) Å
*b* = 9.7485 (10) Å
*c* = 9.8243 (12) Å
β = 90.265 (11)°
*V* = 1232.5 (2) Å^3^
*Z* = 4
Mo *K*α radiation
μ = 0.51 mm^−1^
*T* = 292 K
0.42 × 0.28 × 0.19 mm

#### Data collection

Oxford Diffraction Xcalibur diffractometer with an Eos (Nova) detector
Absorption correction: multi-scan (*CrysAlis Pro*; Oxford Diffraction, 2009 ▶) *T*_min_ = 0.815, *T*_max_ = 0.910
13416 measured reflections
2407 independent reflections
1678 reflections with *I* > 2σ(*I*)
*R*_int_ = 0.041

#### Refinement

*R*[*F* ^2^ > 2σ(*F* ^2^)] = 0.038
*wR*(*F* ^2^) = 0.104
*S* = 1.03
2407 reflections
155 parameters
H-atom parameters constrained
Δρ_max_ = 0.16 e Å^−3^
Δρ_min_ = −0.21 e Å^−3^

### 

Data collection: *CrysAlis Pro* (Oxford Diffraction, 2009 ▶); cell refinement: *CrysAlis Pro*; data reduction: *CrysAlis Pro*; program(s) used to solve structure: *SHELXS97* (Sheldrick, 2008 ▶); program(s) used to refine structure: *SHELXL97* (Sheldrick, 2008 ▶); molecular graphics: *ORTEP-3 for Windows* (Farrugia, 1997 ▶) and *CAMERON* (Watkin *et al.*, 1993 ▶); software used to prepare material for publication: *PLATON* (Spek, 2009 ▶).

## Supplementary Material

Crystal structure: contains datablocks global, I. DOI: 10.1107/S1600536809034308/bg2292sup1.cif

Structure factors: contains datablocks I. DOI: 10.1107/S1600536809034308/bg2292Isup2.hkl

Additional supplementary materials:  crystallographic information; 3D view; checkCIF report

## Figures and Tables

**Table 1:** Hydrogen-bond geometry (Å, °)

*D*—H⋯*A*	*D*—H	H⋯*A*	*D*⋯*A*	*D*—H⋯*A*
N1—H1*N*⋯O1^i^	0.86	2.05	2.883 (2)	163
C13—H13⋯O1	0.93	2.34	2.868 (3)	116

Symmetry code: (i) 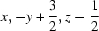.

**Fig. 1.**
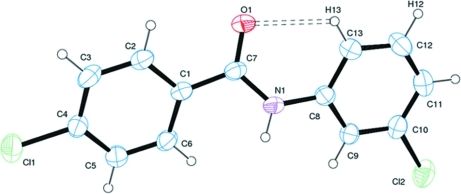
: Molecular structure shows the atom labelling Scheme with displacement ellipsoids for non-H atoms at 50% probability level, hydrogen atoms are arbitary circle. The dotted line shows the C—H···O intramolecular interactions.

**Fig. 2.**
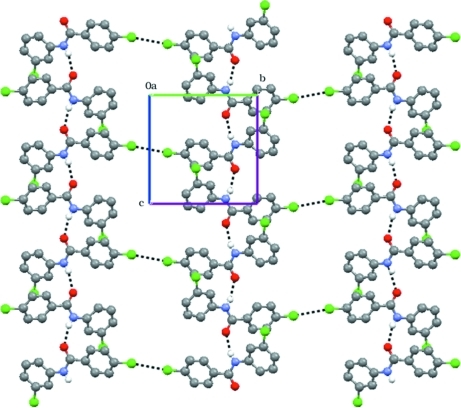
: The molecular packing shows the infinite chain of N—H···O hydrogen bonds along c axis and Cl···Cl interactions as a linker between the chains.

## supplementary crystallographic information

### Comment

The Structure of 4-chloro-*N*-(3-chlorophenyl) benzamide is an extension of our previous work to evaluate the importance of interactions involving halogens in the benzanilide series (Chopra & Guru Row, 2005, 2008). The molecular structure prefers the N—H group to be *trans* to the CO group resulting in the formation of a C—H···O intramolecular interaction (Fig. 1, Table 1) similar to those found in the flourine compounds (Chopra & Guru Row, 2008; Saeed *et al.*, 2008) (Figure 1). The NHCO group forms dihedral angles of 20.2 (2)and 21.5 (1)° with the aniline and benzoyl rings respectively. The two rings are nearly coplanar, with dihedral angle of 3.7 (2)°. The crystal packing is formed by infinite chains with N—H···O hydrogen bonds along the *c* axis (Figure 2, Table 1). Similar interactions were observed in the analogous chloro substituted benzanilides (Gowda *et al.*, 2008; Saeed *et al.*, 2008). There is a halogen Cl1···Cl1^i^ contact (i): -*x* + 1, -*y* + 2, -*z, *(3.47 (1) Å, [Type-I, θ_1_=θ_2_=171.1 (2)°] (Bui *et al.*, 2009) which links chains across an inversion centre, (Figure 2). In addition π···π stacking enhance the stability of the packing across the centres of symmetry .(*Cg*_1_···*Cg*_1_^ii^_=_ 3.71 (2)Å, *Cg*_2_···*Cg*_2_^iii^ = 3.77 (2)Å] ; (ii): 1-x,1-y,1-z ; (iii): -x,2-y,1-z; *Cg*1: centroid of the C1—>C6 ring; Cg2: centroid of the C8—>C13 ring).

### Experimental

The title compound (Scheme) was prepared according to the literature method (Chopra & Guru Row, 2005). The purity of the compound was confirmed by infrared and NMR spectra. Single crystals were grown from ethanol at room temperature and used for X-ray diffraction study.

### Refinement

All H atoms were positioned geometrically, (C—H = 0.93 Å, N—H = 0.86 Å) and refined using a riding model with *U*_iso_(H)= 1.2 *U*_eq_(C, N). The crystal used for data collection was a twin, with twin law 1 0 0, 0 1 0, 0 0 1, as disclosed by ROTAX, Pearson & Gould (2003), and confirmed by refinement with the TWIN instruction in SHELXL97, Sheldrick (2008), leading to a distribution (BASF parameter) of 0.948/0.052 (1).

### Crystal data

**Table:**

C_13_H_9_Cl_2_NO	*F*(000) = 544
*M**_r_* = 266.11	*D*_x_ = 1.434 Mg m^−^^3^
Monoclinic, *P*2_1_/*c*	Mo *K*α radiation, λ = 0.7107 Å
Hall symbol: -P 2ybc	Cell parameters from 350 reflections
*a* = 12.8696 (15) Å	θ = 1.0–28.0°
*b* = 9.7485 (10) Å	µ = 0.51 mm^−^^1^
*c* = 9.8243 (12) Å	*T* = 292 K
β = 90.265 (11)°	Plate, colorless
*V* = 1232.5 (2) Å^3^	0.42 × 0.28 × 0.19 mm
*Z* = 4	

### Data collection

**Table:**

Oxford Diffraction Xcalibur diffractometer with an Eos (Nova) detector	2407 independent reflections
Radiation source: Enhance (Mo) X-ray Source	1678 reflections with *I* > 2σ(*I*)
graphite	*R*_int_ = 0.041
Detector resolution: 16.0839 pixels mm^-1^	θ_max_ = 26.0°, θ_min_ = 3.2°
ω scans	*h* = −15→15
Absorption correction: multi-scan (*CrysAlis PRO*; Oxford Diffraction, 2009)	*k* = −12→12
*T*_min_ = 0.815, *T*_max_ = 0.910	*l* = −12→12
13416 measured reflections	

### Refinement

**Table:**

Refinement on *F*^2^	Primary atom site location: structure-invariant direct methods
Least-squares matrix: full	Secondary atom site location: difference Fourier map
*R*[*F*^2^ > 2σ(*F*^2^)] = 0.038	Hydrogen site location: inferred from neighbouring sites
*wR*(*F*^2^) = 0.104	H-atom parameters constrained
*S* = 1.03	*w* = 1/[σ^2^(*F*_o_^2^) + (0.0559*P*)^2^] where *P* = (*F*_o_^2^ + 2*F*_c_^2^)/3
2407 reflections	(Δ/σ)_max_ = 0.001
155 parameters	Δρ_max_ = 0.16 e Å^−^^3^
0 restraints	Δρ_min_ = −0.21 e Å^−^^3^

### Special details

**Table:**

Geometry. All e.s.d.'s (except the e.s.d. in the dihedral angle between two l.s. planes) are estimated using the full covariance matrix. The cell e.s.d.'s are taken into account individually in the estimation of e.s.d.'s in distances, angles and torsion angles; correlations between e.s.d.'s in cell parameters are only used when they are defined by crystal symmetry. An approximate (isotropic) treatment of cell e.s.d.'s is used for estimating e.s.d.'s involving l.s. planes.
Refinement. Refinement of *F*^2^ against ALL reflections. The weighted *R*-factor *wR* and goodness of fit *S* are based on *F*^2^, conventional *R*-factors *R* are based on *F*, with *F* set to zero for negative *F*^2^. The threshold expression of *F*^2^ > σ(*F*^2^) is used only for calculating *R*-factors(gt) *etc*. and is not relevant to the choice of reflections for refinement. *R*-factors based on *F*^2^ are statistically about twice as large as those based on *F*, and *R*- factors based on ALL data will be even larger.

### Fractional atomic coordinates and isotropic or equivalent isotropic displacement parameters (Å^2^)

**Table:**

	*x*	*y*	*z*	*U*_iso_*/*U*_eq_	
Cl2	−0.01097 (5)	1.07010 (7)	0.17348 (7)	0.0830 (3)	
Cl1	0.45841 (6)	0.16654 (6)	0.53117 (7)	0.0811 (3)	
O1	0.27955 (14)	0.79207 (16)	0.68009 (14)	0.0682 (5)	
N1	0.24298 (14)	0.79496 (18)	0.45618 (16)	0.0512 (4)	
H1N	0.2531	0.7516	0.3812	0.061*	
C7	0.28063 (16)	0.7347 (2)	0.56910 (19)	0.0485 (5)	
C1	0.32291 (15)	0.5931 (2)	0.55447 (18)	0.0459 (5)	
C9	0.12551 (16)	0.9361 (2)	0.3325 (2)	0.0513 (5)	
H9	0.1205	0.8662	0.2683	0.062*	
C8	0.18891 (16)	0.9205 (2)	0.44516 (19)	0.0464 (5)	
C6	0.29668 (18)	0.5044 (2)	0.4485 (2)	0.0561 (6)	
H6	0.2510	0.5339	0.3810	0.067*	
C2	0.39013 (18)	0.5444 (2)	0.6539 (2)	0.0599 (6)	
H2	0.4077	0.6010	0.7267	0.072*	
C13	0.19596 (19)	1.0256 (2)	0.5399 (2)	0.0602 (6)	
H13	0.2385	1.0167	0.6162	0.072*	
C3	0.43165 (19)	0.4141 (2)	0.6475 (2)	0.0634 (6)	
H3	0.4773	0.3836	0.7147	0.076*	
C4	0.40478 (17)	0.3300 (2)	0.5410 (2)	0.0559 (6)	
C5	0.33695 (18)	0.3742 (2)	0.4418 (2)	0.0602 (6)	
H5	0.3185	0.3162	0.3705	0.072*	
C11	0.0749 (2)	1.1604 (2)	0.4082 (3)	0.0699 (7)	
H11	0.0364	1.2403	0.3963	0.084*	
C10	0.06962 (18)	1.0554 (2)	0.3151 (2)	0.0577 (6)	
C12	0.1386 (2)	1.1438 (3)	0.5190 (3)	0.0748 (7)	
H12	0.1434	1.2143	0.5825	0.090*	

### Atomic displacement parameters (Å^2^)

**Table:**

	*U*^11^	*U*^22^	*U*^33^	*U*^12^	*U*^13^	*U*^23^
Cl2	0.0831 (5)	0.0747 (5)	0.0911 (5)	0.0067 (3)	−0.0221 (4)	0.0229 (3)
Cl1	0.0923 (5)	0.0617 (4)	0.0892 (5)	0.0194 (3)	−0.0156 (4)	0.0071 (3)
O1	0.1067 (13)	0.0635 (9)	0.0343 (8)	0.0055 (9)	0.0007 (8)	−0.0038 (7)
N1	0.0644 (11)	0.0550 (10)	0.0343 (9)	0.0082 (9)	−0.0023 (8)	−0.0031 (7)
C7	0.0550 (13)	0.0561 (13)	0.0344 (11)	−0.0050 (10)	0.0026 (9)	0.0013 (10)
C1	0.0491 (12)	0.0533 (13)	0.0351 (10)	−0.0028 (10)	−0.0013 (9)	0.0045 (9)
C9	0.0556 (13)	0.0462 (12)	0.0520 (13)	−0.0034 (10)	0.0010 (10)	0.0026 (9)
C8	0.0492 (12)	0.0462 (12)	0.0439 (11)	−0.0008 (9)	0.0080 (9)	0.0015 (9)
C6	0.0640 (14)	0.0567 (14)	0.0475 (12)	0.0033 (11)	−0.0149 (11)	0.0039 (10)
C2	0.0721 (15)	0.0671 (15)	0.0402 (12)	0.0010 (12)	−0.0125 (11)	0.0003 (10)
C13	0.0698 (15)	0.0545 (14)	0.0562 (13)	−0.0013 (12)	−0.0024 (12)	−0.0046 (11)
C3	0.0696 (15)	0.0708 (16)	0.0497 (13)	0.0078 (12)	−0.0142 (12)	0.0146 (11)
C4	0.0595 (14)	0.0525 (13)	0.0557 (13)	0.0053 (10)	−0.0036 (11)	0.0106 (10)
C5	0.0723 (15)	0.0546 (14)	0.0536 (13)	0.0021 (12)	−0.0141 (11)	−0.0016 (11)
C11	0.0802 (17)	0.0479 (14)	0.0816 (18)	0.0108 (12)	0.0045 (15)	0.0070 (12)
C10	0.0582 (14)	0.0512 (13)	0.0638 (14)	0.0002 (11)	0.0019 (11)	0.0132 (11)
C12	0.096 (2)	0.0515 (15)	0.0774 (17)	0.0021 (14)	0.0097 (16)	−0.0137 (13)

### Geometric parameters (Å, °)

**Table:**

Cl2—C10	1.738 (2)	C6—H6	0.9300
Cl1—C4	1.740 (2)	C2—C3	1.380 (3)
O1—C7	1.226 (2)	C2—H2	0.9300
N1—C7	1.344 (2)	C13—C12	1.384 (3)
N1—C8	1.412 (3)	C13—H13	0.9300
N1—H1N	0.8600	C3—C4	1.373 (3)
C7—C1	1.491 (3)	C3—H3	0.9300
C1—C2	1.385 (3)	C4—C5	1.375 (3)
C1—C6	1.394 (3)	C5—H5	0.9300
C9—C10	1.378 (3)	C11—C12	1.369 (4)
C9—C8	1.381 (3)	C11—C10	1.374 (3)
C9—H9	0.9300	C11—H11	0.9300
C8—C13	1.386 (3)	C12—H12	0.9300
C6—C5	1.372 (3)		
			
C7—N1—C8	128.18 (17)	C12—C13—C8	118.8 (2)
C7—N1—H1N	115.9	C12—C13—H13	120.6
C8—N1—H1N	115.9	C8—C13—H13	120.6
O1—C7—N1	121.9 (2)	C4—C3—C2	119.22 (19)
O1—C7—C1	120.94 (18)	C4—C3—H3	120.4
N1—C7—C1	117.11 (17)	C2—C3—H3	120.4
C2—C1—C6	117.5 (2)	C3—C4—C5	120.7 (2)
C2—C1—C7	118.44 (18)	C3—C4—Cl1	119.41 (17)
C6—C1—C7	124.01 (17)	C5—C4—Cl1	119.90 (18)
C10—C9—C8	119.9 (2)	C6—C5—C4	119.6 (2)
C10—C9—H9	120.1	C6—C5—H5	120.2
C8—C9—H9	120.1	C4—C5—H5	120.2
C9—C8—C13	119.6 (2)	C12—C11—C10	117.9 (2)
C9—C8—N1	116.49 (18)	C12—C11—H11	121.0
C13—C8—N1	123.91 (19)	C10—C11—H11	121.0
C5—C6—C1	121.30 (19)	C11—C10—C9	121.5 (2)
C5—C6—H6	119.4	C11—C10—Cl2	119.93 (18)
C1—C6—H6	119.4	C9—C10—Cl2	118.53 (18)
C3—C2—C1	121.6 (2)	C11—C12—C13	122.3 (2)
C3—C2—H2	119.2	C11—C12—H12	118.9
C1—C2—H2	119.2	C13—C12—H12	118.9
			
C8—N1—C7—O1	−6.8 (3)	C9—C8—C13—C12	0.2 (3)
C8—N1—C7—C1	172.39 (18)	N1—C8—C13—C12	−179.4 (2)
O1—C7—C1—C2	−20.6 (3)	C1—C2—C3—C4	−0.8 (4)
N1—C7—C1—C2	160.2 (2)	C2—C3—C4—C5	−0.3 (4)
O1—C7—C1—C6	157.4 (2)	C2—C3—C4—Cl1	179.05 (18)
N1—C7—C1—C6	−21.8 (3)	C1—C6—C5—C4	−0.2 (3)
C10—C9—C8—C13	−0.2 (3)	C3—C4—C5—C6	0.8 (4)
C10—C9—C8—N1	179.50 (18)	Cl1—C4—C5—C6	−178.57 (18)
C7—N1—C8—C9	−156.2 (2)	C12—C11—C10—C9	0.6 (4)
C7—N1—C8—C13	23.4 (3)	C12—C11—C10—Cl2	179.3 (2)
C2—C1—C6—C5	−0.8 (3)	C8—C9—C10—C11	−0.3 (3)
C7—C1—C6—C5	−178.9 (2)	C8—C9—C10—Cl2	−178.96 (16)
C6—C1—C2—C3	1.3 (3)	C10—C11—C12—C13	−0.6 (4)
C7—C1—C2—C3	179.5 (2)	C8—C13—C12—C11	0.1 (4)

### Hydrogen-bond geometry (Å, °)

**Table:**

*D*—H···*A*	*D*—H	H···*A*	*D*···*A*	*D*—H···*A*
N1—H1N···O1^i^	0.86	2.05	2.883 (2)	163
C13—H13···O1	0.93	2.34	2.868 (3)	116

Symmetry codes: (i) *x*, −*y*+3/2, *z*−1/2.
